# The Prognostic Importance of Right Ventricular Longitudinal Strain in Patients with Cardiomyopathies, Connective Tissue Diseases, Coronary Artery Disease, and Congenital Heart Diseases

**DOI:** 10.3390/diagnostics11060954

**Published:** 2021-05-26

**Authors:** Marijana Tadic, Johannes Kersten, Nicoleta Nita, Leonhard Schneider, Dominik Buckert, Birgid Gonska, Dominik Scharnbeck, Tilman Dahme, Armin Imhof, Evgeny Belyavskiy, Cesare Cuspidi, Wolfgang Rottbauer

**Affiliations:** 1 Klinik für Innere Medizin II, Universitätsklinikum Ulm, Albert-Einstein Allee 23, 89081 Ulm, Germany; johannes.kersten@uni-ulm.de (J.K.); Nicoleta.Nita@uniklinik-ulm.de (N.N.); Leonhard.Schneider@uniklinik-ulm.de (L.S.); dominik.buckert@uniklinik-ulm.de (D.B.); Birgid.Gonska@uniklinik-ulm.de (B.G.); Dominik.Scharnbeck@uniklinik-ulm.de (D.S.); Tilman.Dahme@uniklinik-ulm.de (T.D.); Armin.Imhof@uniklinik-ulm.de (A.I.); wolfgang.rottbauer@uniklinik-ulm.de (W.R.); 2 Department of Cardiology, Charité—Universitätsmedizin Berlin (Campus Virchow-Klinikum), 13353 Berlin, Germany; Evgeny.Belyavskiy@charite.com; 3 Department of Medicine and Surgery, University of Milan-Bicocca, 20126 Milan, Italy; cesare.cuspidi@unimib.com

**Keywords:** right ventricle, strain, echocardiography, magnetic resonance, cardiomyopathy, amyloidosis, systemic sclerosis, lupus, myocardial infarction

## Abstract

Right ventricular (RV) systolic function represents an important independent predictor of adverse outcomes in many cardiovascular (CV) diseases. However, conventional parameters of RV systolic function (tricuspid annular plane excursion (TAPSE), RV myocardial performance index (MPI), and fractional area change (FAC)) are not always able to detect subtle changes in RV function. New evidence indicates a significantly higher predictive value of RV longitudinal strain (LS) over conventional parameters. RVLS showed higher sensitivity and specificity in the detection of RV dysfunction in the absence of RV dilatation, apparent wall motion abnormalities, and reduced global RV systolic function. Additionally, RVLS represents a significant and independent predictor of adverse outcomes in patients with dilated cardiomyopathy (CMP), hypertrophic CMP, arrhythmogenic RV CMP, and amyloidosis, but also in patients with connective tissue diseases and patients with coronary artery disease. Due to its availability, echocardiography remains the main imaging tool for RVLS assessment, but cardiac magnetic resonance (CMR) also represents an important additional imaging tool in RVLG assessment. The findings from the large studies support the routine evaluation of RVLS in the majority of CV patients, but this has still not been adopted in daily clinical practice. This clinical review aims to summarize the significance and predictive value of RVLS in patients with different types of cardiomyopathies, tissue connective diseases, and coronary artery disease.

## 1. Introduction

The assessment of right ventricular (RV) function may be challenging due to the anatomical shape and position of the RV. A large part of the RV is difficult to visualize by echocardiographic examination, particularly for two-dimensional echocardiography. Several echocardiographic views are necessary to assess RV structure and function, which significantly limits the feasibility of complete evaluation of global and regional contractile function. The apical 4-chamber echocardiographic view is the most used window that allows the assessment of several important quantitative parameters [1].

RV systolic function was proven to be an important independent predictor of adverse outcomes in a large number of cardiovascular (CV) diseases and new evidence indicated a higher predictive value of RV longitudinal strain (LS) over conventional parameters of RV systolic function (tricuspid annular plane excursion (TAPSE), systolic flow velocity across the lateral segment of tricuspid annulus obtained by tissue Doppler (s’), RV myocardial performance index (MPI), and fractional area change (FAC)) [2,3,4]. RV free wall LS showed higher sensitivity and specificity in the detection of RV dysfunction in the absence of RV dilatation, wall motion abnormalities, and a reduced global RV systolic function [5]. Moreover, RVLS was a significant and independent predictor of outcome (clinical worsening—hospitalizations and mortality) [2,3,4,5]. This has recently been reported in patients with dilated cardiomyopathy (CMP), hypertrophic CMP, arrhythmogenic RV CMP and, amyloidosis [3,4,5,6]. Similar results of the significant predictive value of RV free wall LS were found in patients with connective tissue diseases and patients with coronary artery disease [7,8]. Even though echocardiography remains the main imaging tool for RVLS assessment, cardiac magnetic resonance (CMR) became an important additional source of RVLG information in patients with different indications, primarily various CMPs [9,10,11]. These findings support the routine evaluation of RVLS in the majority of CV patients. However, this has not yet been adopted in the majority of echocardiographic and CMR laboratories.

This review article aims to provide a clinical summary of the importance and predictive value of RVLS in patients with different cardiomyopathies, tissue connective diseases, and coronary artery disease.

## 2. Cardiomyopathies

**Figure 1:**
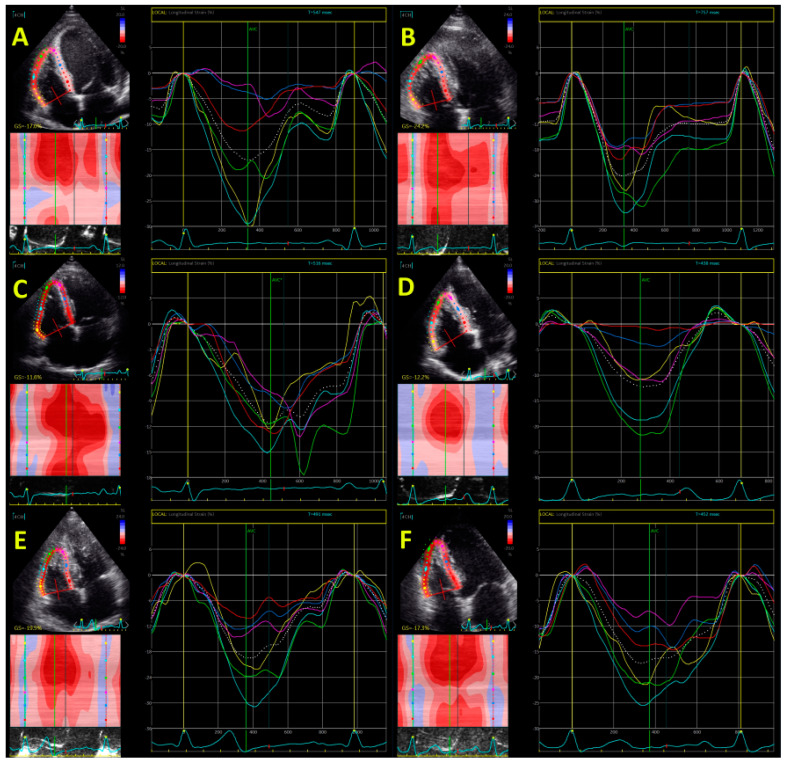
Echocardiography-derived right ventricular global longitudinal strain in patient with dilated cardiomyopathy (panel **A**); hypertrophic cardiomyopathy (panel **B**); arrhythmogenic right ventricular cardiomyopathy (panel **C** and **D**); amyloidosis (panel **E**); and acute myocardial infarction (panel **F**).

RV systolic function has a very important role in patients with CMPs. TAPSE, s’, and FAC have been long used as the only parameters of RV systolic function and they demonstrated a significant predictive value in patients with dilated, hypertrophic, and arrhythmogenic CMP, as well as in cardiac amyloidosis. The introduction of speckle tracking echocardiographic imaging significantly changed the perception of RV systolic function evaluation and particularly when studies showed its advantages in the prediction of adverse outcomes in these patients. Echocardiography has a pivotal role in the RV assessment and particularly in evaluation of RVLS. However, due to its possibility for accurate estimation of RV volumes, RV ejection fraction (RVEF), as well as left ventricular (LV) tissue characterization, CMR became more utilized over the last decade. Figure 1 shows echocardiographic assessment of RVLS in patients with different cardiomyopathies.

### 2.1. Dilative Cardiomyopathy

The initial investigations using echocardiography-derived RVLS showed significantly lower values of RV global LS and free-wall LS in patients with idiopathic DCM, whereas TAPSE, s’, RV MPI, and FAC could not identify any difference between idiopathic and ischemic DCM patients [12]. A small study that included 40 patients with non-ischemic DCM showed that all parameters of RV systolic function—TAPSE, s’, RV MPI, and RV free-wall LS—were independent predictors of major CV event (death, hospitalization for decompensation heart disease, and the occurrence of ventricular arrhythmia) in the period of 6 months [13]. Another investigation aimed to determine the short-term effect of levosimendan on LV and RV functions in patients with non-ischemic DCM and reported that all parameters of RV systolic function (TAPSE, RV MPI, and RVLS) significantly improved after 72 h and maintained (to a lesser extent) even 1 month after levosimendan therapy [14]. Table 1 summarizes studies about RVLS in DCM patients.

Seo et al. involved 143 patients with DCM in sinus rhythm and followed them for 40 months and showed that there was no difference in the conventional parameters of LV and RV function between patients who experience primary endpoint and those who did not [15]. The primary endpoints were defined as all-cause mortality, hospitalization due to heart failure deteriorating, and fatal arrhythmia or aborted sudden cardiac death [15]. Multivariate analysis showed that only RV free-wall LS was associated with adverse outcome independently of conventional parameters of LV and RV function [15]. The investigators proposed the certain cut-off values for each RV parameter of systolic function (Table 1) [15]. Marsumoto et al. included 104 patients with DCM who were followed for 17 months and reported significant predictive value of RV contractile reserve, assessed during the dobutamine stress [9]. Namely, RV contractile reserve, assessed only by improvement of RVLS, was an independent predictor of adverse CV outcome in patients with DCM [9]. Interestingly, parameters of LV contractile reserve that showed an independent predictive role were change in LV circumferential strain and LVEF during dobutamine test, but not improvement in LVLS [9].

There is lack of agreement about the predictive value of RVLS in DCM. Monaghetti et al. investigated 208 patients with DCM and none of the RV systolic function parameters (TAPSE, FAC, or RVLS) was an independent predictor of composite outcome (death, heart transplant, LV device implantation, and hospitalization for acute heart failure) [17]. The only independent predictors were NYHA class, LVEF, and functional capacity m6asured by peak VO2 [16].

**Figure 2:**
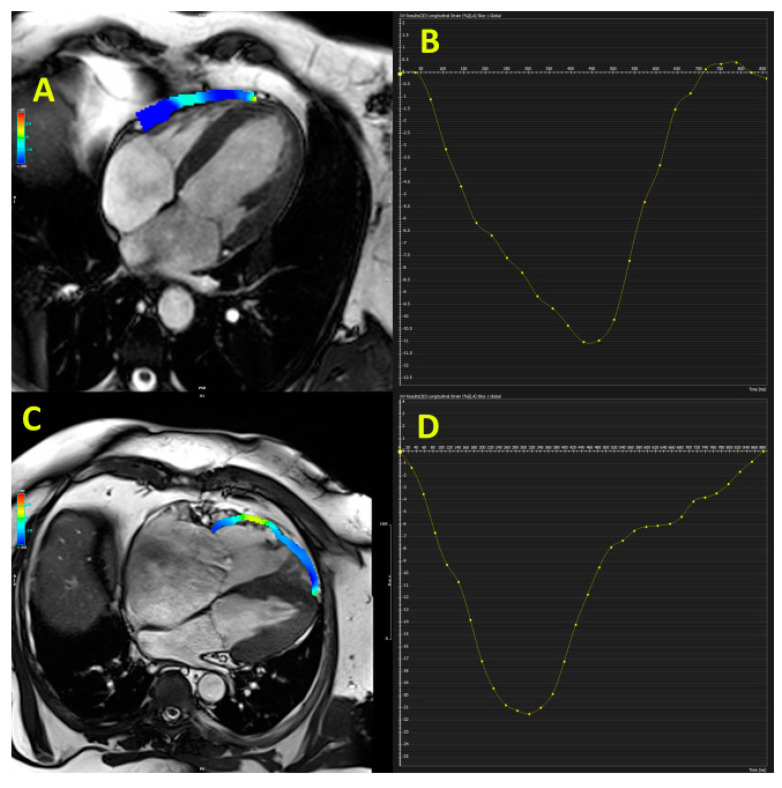
Cardiac magnetic resonance-derived right ventricular free-wall longitudinal strain at early stage of dilated cardiomyopathy (panel **A** and **B**) and patient with arrhythmogenic right ventricular cardiomyopathy (panel **C** and **D**).

CMR analyses reported a significant prognostic impact of RVLS in patients with DCM. The investigation that involved 192 patients with non-ischemic DCM who were followed for 5 years showed that RVLS ≥ −8.5% was independently associated with the composite outcome (major adverse cardiac events (MACEs), including CV death and cardiac transplantation) [17]. The large investigation that included 441 patients with non-ischemic DCM reported that CMR-derived RV free-wall LS during 4.2-year follow-up was an independent predictor of the primary endpoint (cardiac death and cardiac transplantation) and combined endpoint (primary endpoint plus occurrence of ventricular tachycardia/fibrillation or hospitalization due to congestive heart failure) [9]. Figure 1 (panel A) shows reduced echocardiography-derived RVLS in patients with DCM and Figure 2 illustrates preserved RV free-wall strain in patient with early stage of DCM (panel A and B). These findings confirm that RVLS provides incremental information beyond clinical and standard RV parameters and therefore may be used for better risk stratification in DCM patients.

### 2.2. Hypertrophic Cardiomyopathy

RV involvement in patients with hypertrophic cardiomyopathy (HCM) consists of structural and functional changes. The incidence of RV involvement in HCM patients varies significantly depending on the criteria and the method used for the assessment of RV structure and function. The histopathological characteristics of HCM are general myocyte hypertrophy, regions of myocyte disarray, different types of fibrosis, and small-vessel disease [18,19]. In the majority of patients, myocyte disarray is extensive and involves more than 20% of the myocardium in at least two tissue blocks [20]. CMR studies reported RV hypertrophy in 30% [21], whereas echocardiographic investigation identified RV hypertrophy in 44% of HCM patients [20]. Conventional parameters of RV systolic function (TAPSE, FAC, and s’) often remain within normal limits, which does not facilitate the detection of RV subclinical impairment in HCM patients [22]. The same investigation revealed a significant reduction in RV global LS and free-wall LS in HCM patients with RV hypertrophy, but not in those without RV hypertrophy [22]. Only RV thickness, but not LS or s’, was independently associated with arrhythmias in HCM patients.

D’Andrea et al. used RVLS to distinguish HCM patients from professional athletes and suggested a cut-off value for RVLS of >−16% [23]. The same group of authors showed that increases of TAPSE and s’ during physical effort were similar between HCM patients and controls [24]. However, the increase of RV free-wall LS was significantly lower in HCM patients than in controls. The multivariable analysis detected independent associations of RV lateral strain at peak stress with the maximal workload during exercise. Cut-off for RV free-wall LS of −14% differentiated controls and HCM [25]. Another study reported that RVLS was significantly impaired in HCM compared with hypertensive patients and healthy controls and proposed a cut-off value for RVLS of > −14.9% to diagnose HCM individuals [26]. Investigation that studied HCM patients with and without LV outflow (LVOT) obstruction showed significantly reduced LV and RVLS in HCM patients compared with control groups, but there was no difference between patients with and without LVOT obstruction [25]. Figure 1 (panel B) demonstrates echocardiography-derived RVLS in HCM patient.

The only echocardiographic study that investigated the predictive value of RVLS in HCM patients included 267 HCM patients and reported that patients with RV global LS ≥ −20% and those with RV free-wall LS > −19% had significantly higher mortality during 6.7-year follow-up [27]. Multivariable analysis showed that RV global LS was independently associated with endpoint (all-cause mortality and development of heart failure) [27]. Table 2 summarizes investigations about the predictive value of RVLS in HCM patients.

CMR study showed that RV longitudinal, circumferential, and radial strains were significantly lower in HCM patients than in controls [28]. All types of RV strains correlated well with corresponding LV strains. Similar to the echocardiographic studies, the CMR study revealed that RV global and apical LS in HCM patients with RV hypertrophy were significantly lower than in HCM patients without RV hypertrophy and controls [29]. The global, apical, and mid-ventricular RVLS in HCM patients with RV late gadolinium enhancement (LGE) were significantly lower than in HCM patients without RV-LGE and controls. No significant difference was found regarding global and regional LS in HCM patients with LVOT obstruction compared to those without LVOT obstruction [29].

Seo et al. performed echocardiographic and CMR studies in 256 HCM patients and provided a 3-year follow-up [4]. The authors defined RV involvement as RV wall thickness ≥7 mm and reported that 14.4% of patients had RV involvement. RV involvement was independently associated with primary outcomes (all-cause death, heart transplantation, and unplanned cardiovascular admission). Patients with RV hypertrophy had significantly more impaired RV strain, which was independently associated with primary outcomes [4]. RV hypertrophy in HCM patients was associated with more advanced LV structure and biventricular dysfunction, indicating severe HCM. RV hypertrophy and reduced echocardiography-derived RV free-wall LS (>−20%) had a prognostic value related to clinical adverse events in HCM patients [4].

Yang et al. included 384 HCM patients and 150 healthy volunteers and showed that RV LS was an independent prognostic factor for the primary and secondary endpoints [30]. The primary endpoint included all-cause mortality and sudden cardiac death aborted by appropriate implantable cardioverter-defibrillator discharge and cardiopulmonary resuscitation after syncope. The secondary endpoint was a combination of the primary endpoint and hospitalization due to congestive heart failure [30]. The authors used different RVLS cut-off values for primary and secondary endpoints: > −10.9% and > −12.8%, respectively. In the subgroup of patients with a normal RVEF, impaired RVLS was related to adverse outcomes and might add incremental prognostic value to RVEF and TAPSE. Even though no study compared the impact of CMR-derived late gadolinium enhancement (LGE) and RVLS on outcome in HCM patients, Li et al. reported significantly lower value of RVLS in patients with HCM RV LGE than in those HCM patients without detected LGE [29].

### 2.3. Arrhythmogenic Right Ventricular Cardiomyopathy

Traditional echocardiographic parameters for assessment of RV systolic function such as TAPSE, FAC, and s’ are non-specific and could not provide reliable information about fibro-fatty replacement and kinetic disturbances that are characteristics of arrhythmogenic right ventricular cardiomyopathy (ARVC). RVLS seems to be a reliable measurement of impaired wall motion and has important diagnostic value in the absence of RV dilatation, visual wall motion abnormalities, and a reduced RV systolic function measured by conventional parameters. A cut-off value of −18% for RVLS has been proposed to discriminate normal from abnormal RV segments for diagnosing ARVC patients with high diagnostic accuracy [31]. Figure 1 (panel C and D) shows echocardiography-derived RVLS in two patients with ARVC and Figure 1 (panel C and D) illustrates reduced CMR-derived RV free-wall strain in ARVC patient.

One of the first studies showed significantly decreased RVLS in asymptomatic carriers and ARVC patients with arrhythmia (ventricular tachycardia and fibrillation), whereas FAC was reduced only in symptomatic ARVC patients [32]. Multivariate analysis showed that FAC and RV dispersion, but not RVLS, LVLS, and LVEF, were independent predictors of arrhythmia in ARVC patients [31]. The meta-analysis of echocardiographic data that included 541 ARVC patients showed a significant reduction of all parameters of RV systolic function (TAPSE, FAC, s’ and RVLS) [33]. Table 2 summarizes research about the predictive importance of RVLS in ARVC.

A recent study showed that echocardiography-derived RV global LS was associated with the risk of RV structural progression during a 5-year follow-up [34]. Patients with an RVLS > −20% had a higher risk of RV structural progression. Interestingly, FAC remained unchanged during the follow-up period. The other study investigated the prognostic value of echocardiographic imaging in first-degree relatives of ARVC patients by dividing them into three separate groups depending on the time of delay of peak systolic strain of RV basal free-wall segment [5]. Type I considers normal deformation, type II considers delayed onset, decreased systolic peak, and post-systolic shortening, and type III mean systolic stretching and large post-systolic shortening [5]. Disease progression after 4-year follow-up was reported in only 4% of the relatives with normal RV deformation at baseline (type I) and in 43% of the relatives with abnormal RV deformation at baseline (type II or III) [5]. A similar study that analyzed 120 ARVC patients and mutation-positive family members investigated the effect of RV mechanical dispersion, defined by previous criteria [5], on the occurrence of life-threatening arrhythmias [35]. Adding RV mechanical dispersion to RV deformation patterns significantly improved the prediction of life-threatening ventricular arrhythmias.

**Table 2:** Predictive value of RVLS in patients with hypertrophic and arrythmogenic right ventricular cardiomyopathy.

Reference	Sample Size	RV Freewall/Global GLS Cut-Off	Follow-Up Period (Months)	Imaging Modality	Main Findings
**Hypertrophic cardiomyopathy**					
Hiemstra et al. [27]	267	−20%	80	Echo	RV global LS was independently associated with end point (all-cause mortality and development of heart failure).
Seo et al. [4]	256	−20%	36	Echo + CMR	RV hypertrophy and reduced echocardiography-derived RV free-wall LS had a prognostic value related to clinical adverse events in HCM patients.
Yang et al. [30]	384	−10.9%	90	CMR	RVLS was an independent prognostic factor for the primary and secondary endpoints.
**Arrhythmogenic right ventricular cardiomyopathy**					
Malik et al. [34]	40	−20%	60	Echo	RVLS had a higher risk of RV structural progression.
Pieles et al. [36]	120	−20%	-	Echo	Reduced RVLS, but not TAPSE and s’, was significantly related with ARVC diagnosis.

ARVC—arrhythmogenic right ventricular cardiomyopathy, CMR—cardiac magnetic resonance, LS—longitudinal strain, RV—right ventricle, s’—systolic velocity of the lateral segment of tricuspid annulus, TAPSE—tricuspid annular plane systolic excursion.

Pieles et al. investigated 120 adolescents with suspected ARVC and demonstrated that patients with definite, borderline, and possible ARVC had significantly lower RVLS than in controls [36]. Multivariable risk analysis revealed that reduced RV strain (>−20.4%), but not TAPSE and s’, was significantly related to ARVC diagnosis [36].

The use of CMR-derived RVLS could help in the differentiation between ARVC patients, borderline ARVC, and those with a positive family history of ARVC [37]. RV circumferential and radial strains were not able to discriminate these three groups, but circumferential strain rate at the basal level could provide this information. A study that compared ARVC patients with those who had RVOT arrhythmias and controls revealed significantly lower RV global LS, circumferential, and radial strains in ARVC patients than in the other two groups, whereas there was no difference between RVOT-related arrhythmia patients and controls [38]. The authors used a cut-off of −23.2% for RV global LS to discriminate ARVC patients from controls. Other authors in a similar group of patients found that discriminator for diagnosis of ARVC was a cut-off of −31% for RV longitudinal strain of basal part of free wall [39].

A recent study compared patients with ARVC, RVOT arrhythmia, and Brugada syndrome and found that multidirectional strain (longitudinal, circumferential, and radial) could be used to distinguish these three groups of patients [40]. It is only important which section (basal, medial, or apical) is taken into consideration. Global RVLS provided differentiation of ARVC from the other two groups, whereas basal and medical circumferential and radial RV strains provided discrimination between all three groups of patients, but not simultaneously [40]. There was no single strain parameter that might differentiate three diagnoses at the same time.

Chen et al. recently reported that CMR-derived RV global LS, circumferential, and radial strains were significantly lower in ARVC patients with LVEF < 55% than in those with LVEF > 55%, even though there was no difference in RVEF between these groups [41]. One of the rare studies that used echocardiography- and CMR-derived RV strain in the same patients showed a similar trend of RV strain deterioration in ARVC patients [42]. There was also a significant level of correlation, but intermodality agreement was weak. The authors concluded that RVLS obtained by both techniques may add a significant value in the assessment of RV function in ARVC, but RVLS values obtained by these two methods should not be used interchangeably in everyday practice [42]. Investigations also reported lower LV radial strain in LV segments of myocardium with LGE than in those without LGE and suggested that regional CMR-derived LV myocardial strain improved detection of arrhythmogenic substrate in comparison with LGE [42,43].

### 2.4. Amyloidosis

Amyloidosis considers the extracellular deposition of insoluble fibrils that consists of different serum proteins (amyloid). There are several types of amyloid proteins that may affect this process: light-chain (LC) immunoglobulin, mutant hereditary transthyretin (TTR), wild-type TTR, mutant apolipoprotein AI, amyloid atrial natriuretic peptide localized to the atrium, fibrinogen alpha type, and serum amyloid A protein. The presence of cardiac amyloid deposits differs with the type of amyloidosis: wild-type amyloidosis and hereditary TTR-related amyloidosis (ATTR) are characterized by a high percentage of cardiac involvement, whereas cardiac involvement is present in about 50% of cases of patients with LC amyloidosis (AL). Figure 1 (panel E) illustrates echocardiography-derived RVLS in a patient with amyloidosis.

RV involvement in ATTR is very frequent and previous studies have showed that TAPSE < 14 mm was an independent predictor of MACE defined as death, heart transplantation, and acute heart failure during the 8-month follow-up period [44]. The echocardiographic study that included patients with amyloidosis, HCM, and controls reported significant a reduction of LV and RVLS from controls, across HCM, to those with cardiac amyloidosis [45]. The authors showed that the improvement of RV or LV longitudinal strain, as well as TAPSE, significantly reduced the risk of MACE. This was not the case with FAC that was not associated with the risk of adverse events. However, in multivariate analysis, after adjustment for clinical and other echocardiographic parameters, RVLS did not remain an independent predictor of reduced risk of MACE, unlike LVLS [46]. Another investigation compared RVLS between AL and ATTR patients and reported significantly lower values of RV free-wall LS, as well as its basal and mid segments, in AL patients compared with ATTR subjects [46]. Interestingly, there was no difference in apical RVLS between these two groups. RV strain analysis indicated an apical sparing pattern, similar to previously described LV apical sparing, with a higher difference between apex and basis, which may represent a specific finding in AL patients [46]. The same analysis compared RV parameters between AL, wild type, and hereditary ATTR. S’ did not have any discriminatory role between three types of cardiac amyloidosis, TAPSE, FAC. RV global LS was significantly lower in hereditary ATTR than in AL patients, while RV free-wall LS was gradually reduced from AL, across ATTRwt to hereditary ATTR patients [46]. There was no other RV parameter that was able to discriminate wild type from hereditary ATTR. Data about predictive value of RVLS are presented in Table 3.

The largest amount of data regarding the prognostic value of RVLS are generated from studies regarding AL. Cappelli et al. investigated patients with AL amyloidosis with and without cardiac involvement that was defined by LV wall thickness >12 mm and reported significantly lower values of TAPSE, s’, and RVLS in patients with cardiac involvement [47]. Univariate analysis showed that RV wall thickness, TAPSE, LV, and RVLS were associated with 19-month mortality in AL patients, but multivariate analysis revealed that only reduced RVLS remained an independent predictor of mortality in this group of patients [47]. Another large echocardiographic study that involved 249 AL patients who were followed for 5 years showed that TAPSE, s’, and RVLS (basal and middle segments of RV free wall) can discriminate AL patients with and without cardiac involvement, whereas FAC was not able to do so [48]. Nevertheless, only the systolic strain rate of the middle segment of RV free wall, besides other clinical and echocardiographic parameters, was an independent predictor of mortality in this population [48]. In the subgroup of patients who underwent echocardiographic examination within 3 months of AL diagnosis, no association between RV mechanics and mortality was detected [48]. A recently published large analysis of 136 patients with cardiac AL showed that LALS and RV free-wall LS were independently associated with 5-year survival in multivariable analysis [6]. LALS was the strongest predictor of survival and the combination of LALS and RV free-wall LS had the highest prognostic value [6].

A small study provided a 5.5-year follow-up of 26 AL patients who underwent autologous hematopoietic stem cell transplantation and showed that baseline LV, RV free-wall, and LA longitudinal strains were predictors of overall survival among these patients after stem cell transplantation [51]. The reduction of RV free-wall strain for 1% in absolute value reduced survival by 14%. After adjusting for age and blood pressure, the association remained significant for LA and RV free-wall LS, whereas LVLS was of borderline statistical significance [49].

CMR studies provided valuable information regarding RV mechanics and its their influence on outcome in AL patients. Li et al. involved 87 AL patients with a 21-month follow-up and reported a significant reduction in RV longitudinal, circumferential, and radial strains in AL patients compared with controls [50]. Moreover, multidirectional strain gradually decreased with increments of AL stage by Mayo classification [52]. Multivariate analysis revealed only RVLS and RV LGE as independent predictors of overall mortality during follow-up [50]. A similar study reported not only that RV multidirectional strain (longitudinal, circumferential, and radial) could be used for discrimination between AL patients with and without cardiac involvement, but also between patients with normal and reduced RVEF and those with and without RV LGE [10]. RV LGE, RVLS, and radial strain were predictors of mortality in univariate analysis, but after adjustment for all RV and LV parameters, as well as for the stage of disease, only RV radial strain was an independent predictor of mortality [10]. Wan et al. followed 129 AL patients for 38 months and revealed that almost all parameters of CMR-derived RV systolic function (RVEF, TAPSE, FAC, RV global LS, RV free-wall LS) were independent predictors of all-cause mortality in AL patients [51]. However, RV free-wall LS was a better predictor of all-cause mortality than RVEF, FAC, or RV global LS in AL patients [51].

## 3. Tissue Connective Disease

Connective tissue diseases are associated with autoimmune response, vasculopathy, and fibrosis of the skin and internal organs. This is particularly valid for systemic sclerosis (SSc), which is related to high mortality due to cardiac and pulmonary affection. Vasospasms of smaller vessels and luminal narrowing induce hypoxia and reperfusion injury, which further promotes fibrosis. Conventional echocardiographic parameters are usually not sensitive enough to detect subtle RV changes in patients with tissue connective disease and therefore speckle-tracking technique has a great value to identify subclinical changes before the manifestation of RV failure.

### 3.1. Systemic Sclerosis

Our study group recently showed that conventional parameters of RV systolic function (TAPSE and FAC) were not different between SSc patients and healthy controls, but 3D RVEF and RVLS, global and free-wall, were significantly lower in SSc patients [53]. Moreover, we found that each RV myocardial layer was impacted in SSc patients. The other study showed that RVLS is reduced in SSc patients globally and regionally, which indicated that basal, midventricular, and apical RVLS were significantly lower in the SSc population than in the control group [54]. TAPSE did not show any difference, but FAC was also lower in SSc patients. Many authors explained RVLS reduction in SSc patients by pulmonary hypertension. However, the study that included only SSc patients without pulmonary hypertension also confirmed the reduction of RVLS in these patients in comparison with controls [55]. The investigation that compared patients SSc and idiopathic pulmonary arterial hypertension revealed significantly lower RVLS in SSc patients [52], which confirmed that mechanisms beyond increased pulmonary resistance and afterload are responsible for the deterioration of RV mechanics in SSc patients. The same investigation demonstrated that reduced RVLS (>−13.7%) was an independent predictor of mortality during the 88-month follow-up period [52]. The study was underpowered to show the difference in predictive value between RVLS and conventional parameters of RV systolic function. Hekimsoy et al. found that LS of the apical segment of the RV free wall > −14.48% had 100% specificity for predicting pulmonary hypertension in SSc patients [56]. CMR-derived RV free-wall LS > −26.2% showed a sensitivity of 84% and a specificity of 77% to predict pulmonary hypertension in SSc patients [57].

The effect of specific therapy for pulmonary hypertension in SSc patients can be also detected by speckle tracking. Mecurio et al. showed that 36-week therapy with ambrisentan and sildenafil in SSc patients with pulmonary hypertension significantly improved RVLS, mainly due to improvement of basal and midventricular segments [58]. The investigators found a significant correlation between RVLS improvement and CMR-derived RV mass [58], which proved the significance of routine echocardiographic evaluation of RVLS in SSc patients.

Other reports did not show the predictive value of RVLS in SSc patients. Tennøe et al. reported that only TAPSE, but not RVLS, was an independent predictor of mortality in SSc patients during a 3.3-year follow-up period [59]. One-millimeter decrease of TAPSE increased mortality risk by 9%.

### 3.2. Systemic Lupus Erythematosus

Data about the involvement of RV and particularly the role of speckle tracking in patients with systemic lupus erythematosus (SLE) are scarce. Leal et al. reported a significant deterioration in RVLS among adolescents with SLE [60]. Similar findings were reported in adults with SLE [61]. However, they reported significantly reduced RVLS in basal, middle, and apical segments only in patients with increased pulmonary pressure (>30 mmHg), but not in those with normal pulmonary pressure [61]. Buonaur et al. compared 50 SLE patients with controls using 3D speckle tracking for LV and 2D speckle tracking for RV strain analysis [62]. The authors did not find a difference in TAPSE and s’ between patients and controls, but they also reported significantly lower 3D RVEF and RVLS (septal and free wall) in the SLE group [62].

A recent meta-analysis that summarized findings from the previously mentioned three studies showed significantly reduced RVLS in SLE patients, as well as a significant reduction in LV multidirectional strain (longitudinal, circumferential, and radial) in patients with SLE [63].

CMR study investigated 47 women with SLE and found that only RV circumferential strain and not RVLS could discriminate SLE patients with normal RVEF (>50%) from those patients with reduced RVEF (<50%) [64]. However, both circumferential and longitudinal RV strain could differentiate between SLE patients with positive and negative LV LGE or between SLE patients with and without pulmonary hypertension [64]. RV circumferential strain was independently associated with extracellular volume and RVEF, whereas RVLS was independently related to pulmonary pressure [65]. Follow-up studies on this topic are not available yet.

## 4. Coronary Artery Disease

The available data about RV involvement in patients with coronary artery disease are limited in comparison with information that we have about the LV role. The recent study showed that TAPSE, FAC, and s’ were independent predictors of MACE in both STEMI and NSTEMI patients in the first 30 days after myocardial infarction [66]. The threshold values used for TAPSE, FAC, and s’ were 15.8 mm; 37.5%, and 9.67 cm/s, respectively [66]. Huttin et al. reported significant improvement in almost all parameters of RV systolic function (TAPSE, FAC, and RVLS) 6 months after acute myocardial infarction (AMI) [65]. The only parameter that did not show significant improvement was s’. The study that included the patients with acute inferior STEMI showed that RVLS had better sensitivity and specificity to predict MACE than TAPSE and FAC [8]. The best cut-off value for the prediction of MASE was RVLS > −15.5%. Patients with RVLS > −15.5% showed a significantly lower 5-year survival rate and lower MACE-free survival rate than the control group [8]. The authors claimed that RVLS could be the strongest parameter of RV systolic function in the prognosis of patients with preserved LV function who underwent PCI for acute inferior STEMI. Chang et al. reported that RV free-wall LS > −18% was an independent predictor of 2-year mortality and ventricular arrhythmias in patients with non-acute coronary syndrome [67]. Table 4 summarizes current data about the predictive value of RVLS in AMI patients.

The large study that included 621 AMI patients treated with PCI showed that TAPSE, FAC, and RV free-wall LS were all predictors of worse outcomes during the 2-year follow-up period [68]. After multivariable analysis, only FAC and RVLS independently predicted the composite end-point (all-cause death, re-infarction, hospitalization due to heart failure). Furthermore, RV free-wall LS > −22.1% provided incremental value to clinical information, infarct characteristics, LVED, and FAC [68]. The reduction of RV free-wall LS for 1% in absolute value was associated with an 8% increase of composite end-point risk [68]. The other large investigation that involved 790 patients with AMI who were followed for 2.5 years demonstrated that reduced RVLS was independently associated with adverse outcomes (sudden cardiac death or malignant ventricular arrhythmias) [69]. RVLS > −22% was proved to be superior that TAPSE in the multivariate model. The research that included 520 AMI patients treated with PCI showed that FAC < 35%, TAPSE < 17 mm, RVLS > −17%, s’ < 9.5 cm/s, RVMPI > 0.43, and tricuspid regurgitation velocity >2.8 m/s were strong independent predictors of in-hospital MACE and 1-year mortality [70]. Figure 1 (Panel F) demonstrates echocardiography-derived RVLS in patient after AMI.

The only CMR study that investigated the predictive value of RVLS included 1235 AMI patients who were treated with PCI demonstrated that RV free-wall LS was an independent predictor of outcome (all-cause death, re-infarction, hospitalization due to heart failure) in addition to age, Killip class, and LVLS, whereas RV ischemia was not independently associated with MASE during 1-year follow-up [71]. The reduction of RVLS for 1% in absolute value was responsible for a 5% increase of MACE risk in multivariate analysis [71].

## 5. Congenital Heart Diseases

The prognostic importance of RVLS has been well established in patients with congenital heart diseases. Recently published data showed that free-wall RVLS (> −17%) was the only independent predictor of low functional capacity in patients with repaired tetralogy of Fallot [72]. Study that investigated the influence of pulmonary valve replacement in the similar group of patients revealed no improvement in CMR-derived RV global LS and circumferential strain in comparison to LV strain that significantly improved after the intervention [73]. Timoteo et al. revealed that RVLSn was an independent predictor of arrhythmic events in patients with repaired tetralogy of Fallot [74].

Moceri et al. investigated patients with pulmonary hypertension associated with congenital heart disease with and without Eisenmenger syndrome and found that transverse RV strain was an independent predictor of survival in this group of patients [75]. In patients with systemic RV with dextroposition of the great arteries after the atrial switch procedure was demonstrated that RVLS < −14.2% was a good predictor of RVEF ≥ 45% [76]. In pediatric population was reported that RVLS may predict neo-aortic arch obstruction after Norwood/Sano procedure in children with hypoplastic left heart syndrome [77]. These findings show that RVLS should be used in assessment of patients with congenital heart diseases because it might help in the reclassification of patients according to the risk to develop adverse outcome.

## 6. Conclusions

In patients with different cardiomyopathies, connective tissue disease, and coronary artery disease, RV involvement is frequently seen. Conventional parameters of RV systolic function could not always detect subtle and subclinical RV changes and therefore RV longitudinal strain might help not only in detection but also in stratification of risk of patients with these cardiovascular conditions to develop the adverse event. RV longitudinal strain (global and free-wall) emerged as an independent predictor of adverse events in addition to clinical parameters and LV function and therefore should be routinely evaluated during echocardiographic examination in these patients.

## Figures and Tables

**Table 1:** Predictive value of RVLS in patients with dilative cardiomyopathy.

Reference	Sample Size	RV Freewall/Global GLS Cut-Off	Follow-Up Period (Months)	Imaging Modality	Main Findings
Zairi et al. [13]	40	−12%	6	Echo	TAPSE, s’, RV MPI, and RV free-wall LS were independent predictors of MACE.
Seo et al. [15]	143	−16.5%	40	Echo	RV free-wall LS was associated with adverse outcome independently of LV volumes, LV systolic and diastolic parameters, left atrial volume Index and other RV parameters of systolic function (TAPSE, FAC, s’).
Marsumoto et al. [3]	104	-	17	Echo	RV contractile reserve, assessed only by improvement of RVLS and not TAPSE and FAC, was independent predictor of adverse CV outcome in patients with DCM.
Monaghetti et al. [16]	208	-	64	Echo	None of the RV systolic function parameters (TAPSE, FAC, or RVLS) were independent predictors of composite outcome (all-cause death, heart transplant, LV device implantation, and hospitalization for acute heart failure).
Liu et al. [17]	192	−8.5%	60	CMR	RVLS was independent predictor of MACEs after adjustment for traditional risk factors and CMR variables.
Arenja et al. [9]	441	−10%	50	CMR	RV free-wall LS was a predictor of primary and combined endpoint independently of clinical characteristics, LVEF, RV volume, RVEF, and TAPSE.

CMR—cardiac magnetic resonance, CV—cardiovascular, DCM—dilative cardiomyopathy, FAC—fractional area change, GLS—global longitudinal strain, LVEF—left ventricular, LS—longitudinal strain, MACE—major adverse cardiovascular events (death, hospitalization for decompensation heart disease, and the occurrence of an ventricular arrhythmia), MPI—myocardial performance index, RV—right ventricle, s’—systolic velocity of the lateral segment of tricuspid annulus, TAPSE—tricuspid annular plane systolic excursion.

**Table 3:** Predictive value of RVLS in patients with amyloidosis.

Reference	Sample Size	RV Freewall/Global GLS Cut-Off	Follow-Up Period (Months)	Imaging Modality	Main Findings
Moñivas Palomero et al. [46]	80	-	-	Echo	TAPSE, FAC and RVLS recognized difference only between AL and hereditary ATTR, whereas RV free-wall LS was able to distinguish AL from ATTRwt and hereditary ATTR.
Cappelli et al. [47]	52	-	19	Echo	RVLS remained an independent predictor of mortality in AL patients.
Bellavia et al. [48]	249	-	60	Echo	TAPSE, s’, and RVLS, unlike FAC, can discriminate AL patients with and without cardiac involvement.
Leedy et al. [49]	26	-	66	Echo	The reduction of RV free-wall LS for 1% in absolute value reduced survival for 14%. LA and RV free-wall LS were independent predictors of mortality in AL patients after autologous hematopoietic stem cell transplantation.
Li et al. [50]	87	-	21	CMR	RVLS and RV late gadolinium enhancement (LGE) were independent predictors of overall mortality in AL patients.
Liu et al. [10]	64	-	20	CMR	RV LGE, RVLS and radial strain were predictors of mortality in univariate analysis, but after adjustment only RV radial strain was independent predictor of mortality in AL patients.
Wan et al. [51]	129	-	38	CMR	All parameters of RV systolic function (RVEF, TAPSE, FAC, RV global LS, RV free-wall LS) were independent predictors of all-cause mortality in AL patients. However, RV free-wall LS was a better predictor of allcause mortality than other indexes.

AL—light chain amyloidosis, CMR—cardiac magnetic resonance, CV—cardiovascular, DCM—dilative cardiomyopathy, FAC—fractional area change, LVEF—left ventricular, LS—longitudinal strain, MACE—major adverse cardiovascular events (death, hospitalization for decompensation heart disease, and the occurrence of an ventricular arrhythmia), RV—right ventricle, s’—systolic velocity of the lateral segment of tricuspid annulus, TAPSE—tricuspid annular plane systolic excursion.

**Table 4:** Predictive value of RVLS in patients with coronary artery disease.

Reference	Sample Size	RV Freewall/Global GLS Cut-Off	Follow-Up Period (Months)	Imaging Modality	Main Findings
Park et al. [8]	282	−15.5%	60	Echo	RVLS had better sensitivity and specificity to predict MACE than TAPSE and FAC in AMI patients.
Chang et al. [67]	208	−18%	24	Echo	RV free-wall was an independent predictor of mortality and ventricular arrhythmias in patients with non-acute coronary syndrome.
Antoni et al. [68]	621	−22.1%	24	Echo	FAC and RVLS independently predicted the composite end-point (all-cause death, re-infarction, hospitalization due to heart failure) in AMI patients.
Risum et al. [69]	790	−22%	30	Echo	RVLS was independently associated with adverse outcome (sudden cardiac death or malignant ventricular arrhythmias) in AMI patients. RVLS was proved to be superior that TAPSE in the multivariate model.
Radwan et al. [70]	520	−17%	12	Echo	FAC, TAPSE, RVLS, s’, RVMPI and tricuspid regurgitation velocity >2.8 m/s were strong independent predictors of in-hospital MACE and 1-year mortality in AMI patients.
Stiermaier et al. [71]	1235	-	12	CMR	RV free-wall LS was an independent predictor of outcome in addition to age, Killip class and LVLS, whereas RV ischemia was not independently associated with MACE during 1-year follow-up in AMI patients.

AMI—acute myocardial infarction, CMR—cardiac magnetic resonance, FAC—fractional area change, LS—longitudinal strain, MACE—major adverse cardiovascular events (death, hospitalization for decompensation heart disease, and the occurrence of an ventricular arrhythmia), MPI—myocardial performance index, RV—right ventricle, s’—systolic velocity of the lateral segment of tricuspid annulus, TAPSE—tricuspid annular plane systolic excursion.
